# Rupture of a Hepatic Hemangioma in the Remnant Liver Following Hybrid Extended Left Hemihepatectomy in a Living Liver Donor: A Case Report

**DOI:** 10.70352/scrj.cr.25-0530

**Published:** 2026-02-11

**Authors:** Rei Tsunoda, Hajime Matsushima, Akihiko Soyama, Ayaka Kinoshita, Kazushige Migita, Ayaka Satoh, Shun Nakamura, Fumika Kamehama, Takashi Hamada, Hajime Imamura, Tomohiko Adachi, Susumu Eguchi

**Affiliations:** Department of Surgery, Nagasaki University Graduate School of Biomedical Sciences, Nagasaki, Nagasaki, Japan

**Keywords:** living donor liver transplantation, hepatic hemangioma, rupture, donor hepatectomy

## Abstract

**INTRODUCTION:**

Hepatic hemangiomas have occasionally been identified in donor liver grafts. However, rupture of a hemangioma in the remnant liver following liver graft harvest in a living donor has not been documented.

**CASE PRESENTATION:**

A 41-year-old male underwent hybrid extended left lobe graft harvesting as a living liver donor for his brother, who had hepatocellular carcinoma and alcohol-induced decompensated liver cirrhosis. Preoperative contrast-enhanced CT revealed a 22-mm hemangioma in segment 6 of the donor liver. Postoperatively, the donor experienced a gradual progression of anemia. On POD 3, contrast-enhanced CT demonstrated an intrahepatic hematoma with active contrast extravasation due to rupture of the hemangioma in the right posterior segment, compressing the right portal vein branch. Emergency surgery was performed, and a large subcapsular hematoma was found on the liver surface. After opening the hepatic capsule, hematoma evacuation, bleeding source identification, and hemostasis were performed. The patient had an uneventful recovery and was discharged home on POD 8.

**CONCLUSIONS:**

This is the first case report of hepatic hemangioma rupture in the remnant liver after graft harvest in a living liver donor.

## Abbreviations

ALT
: alanine aminotransferase
AST
: aspartate aminotransaminase
Hb
: hemoglobin
Hct
: hematocrit
IVR
: interventional radiology
LDLT
: living donor liver transplantation
TAE
: transcatheter arterial embolization

## INTRODUCTION

Hepatic hemangioma is the most common benign tumor of the liver, with a reported prevalence of 0.4%–20% in the general population, depending on the diagnostic modality used.^1)^ Although typically asymptomatic and incidentally detected, hepatic hemangioma has a rare but potentially life-threatening risk of rupture. While spontaneous rupture has been reported more frequently in large or giant lesions, several case reports and reviews have indicated that rupture can also occur in smaller lesions in response to certain triggers, including anticoagulation therapy and blunt abdominal trauma.^2–4)^

In the setting of LDLT, hepatic hemangiomas have occasionally been identified in donor liver grafts.^5–7)^ Here, we report the first known case of rupture of a small hepatic hemangioma in the remnant liver following hybrid extended left hemihepatectomy in a living liver donor. We describe the clinical course, the possible mechanisms leading to the rupture, and the rationale for surgical management in this unusual scenario.

## CASE PRESENTATION

**Fig. 1:**
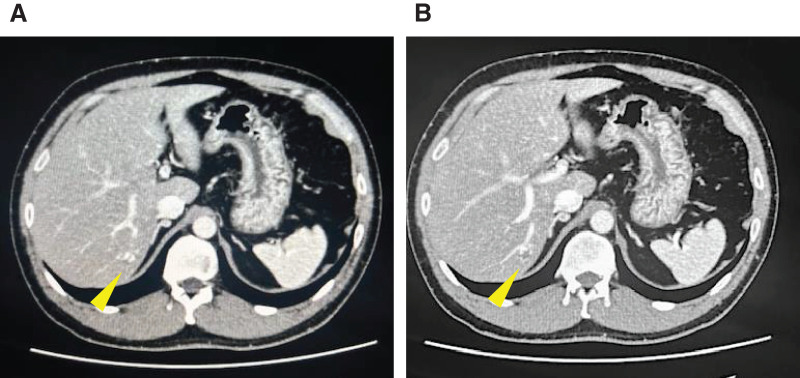
Preoperative contrast-enhanced CT scan. (**A**, **B**) A 22-mm nodule (yellow arrow) was detected in hepatic segment 6, with peripheral enhancement in the arterial phase (**A**) and relatively homogeneous enhancement in the delayed phase (**B**).

**Fig. 2:**
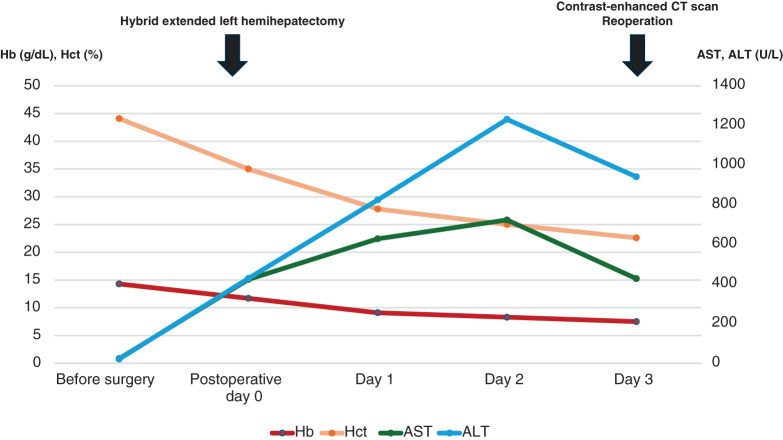
Postoperative trends in laboratory test results. No transfusions were administered during the period before the reoperation.

**Fig. 3:**
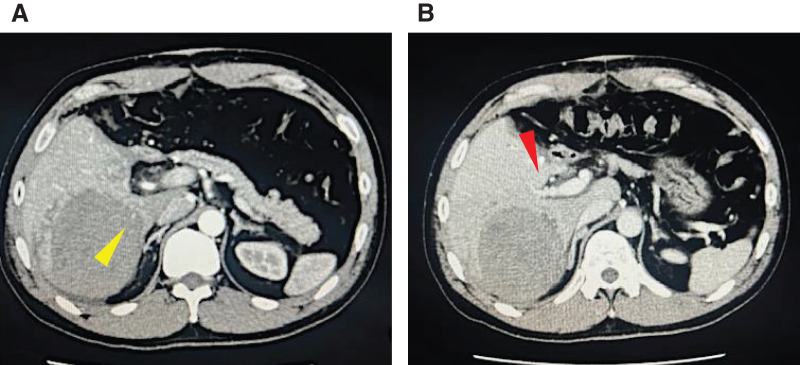
Contrast-enhanced CT scan on POD 3 following extended left hemihepatectomy. (**A**) In the arterial phase, a large hematoma with contrast medium extravasation (yellow arrow) was observed in the posterior segment of the liver. (**B**) In the portal venous phase, the anterior branch of the portal vein (red arrow) was compressed and narrowed by the hematoma.

**Fig. 4:**
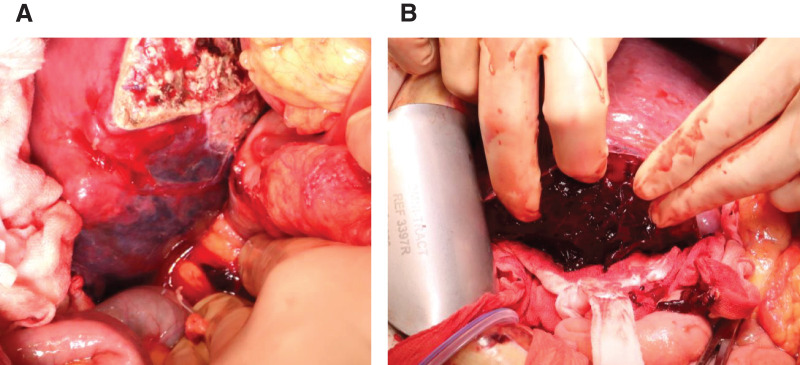
Intraoperative findings. (**A**) On laparotomy, a large intrahepatic hematoma was found in the posterior segment of the liver. (**B**) The hepatic capsule was incised to confirm the hematoma.

**Fig. 5:**
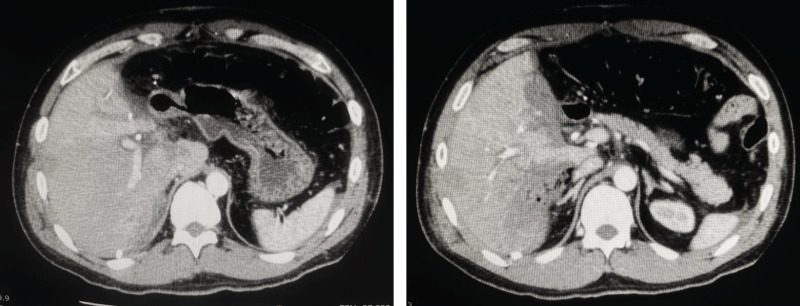
Contrast-enhanced CT images obtained on the day after the reoperation. The hepatic hemangioma was not detected.

A 41-year-old male with no medical history except for Kawasaki disease at 3 years of age was evaluated as a potential living liver donor for his brother, who had hepatocellular carcinoma with decompensated liver cirrhosis due to alcohol-associated liver disease. The results of laboratory tests, including hematological and liver function parameters, were within the normal limits. Preoperative contrast-enhanced abdominal CT detected a 22-mm hemangioma in segment 6 of the liver, which showed gradual peripheral enhancement from the arterial phase to the delayed phase (**Fig. 1**). No vascular anomalies were identified on imaging. An extended left lobe graft with an estimated volume of 520 mL, equivalent to 36.4% of the standard liver volume ratio of the recipient, was selected. A hybrid extended left hemihepatectomy was performed without intraoperative complications, during which hepatic mobilization was accomplished by hand-assisted laparoscopy to the extent that the right adrenal gland was recognized, followed by a parenchymal transection through an upper midline laparotomy using the intermittent Pringle maneuver, as described previously.^8,9)^ The operative time was 426 min, and the intraoperative blood loss was 791 g. The hemangioma was not visualized on the liver surface. Only autologous blood that had been collected preoperatively was transfused intraoperatively. On POD 1, the patient showed mild anemia, but his vital signs remained stable. Because abdominal ultrasonography showed no evidence of intra-abdominal fluid collection and the drainage fluid appeared lightly blood-tinged, the patient was observed conservatively. However, laboratory tests showed further progression of anemia on POD 3 (**Fig. 2**), and a contrast-enhanced CT scan was performed. While no evidence of active bleeding was observed in the peritoneal cavity, we found an intrahepatic hematoma in the right posterior segment of the remnant liver with rupture at the hemangioma, compressing the anterior branch of the portal vein (**Fig. 3**). TAE was considered as an initial treatment to secure hemostasis, but selective embolization of the responsible vessel was considered technically difficult. Furthermore, because the hematoma was compressing the anterior branch of the portal vein, a decision was made to remove the hematoma and achieve surgical hemostasis. On emergency laparotomy, a large subcapsular hepatic hematoma predominantly involving the posterior segment of the liver was found (**Fig. 4**). After the hepatic capsule was incised to evacuate the hematoma, the arterial bleeding point was identified, and hemostasis was achieved by suturing. The operative time was 258 min, and the intraoperative blood loss was 1139 g, including the hematoma. Following the second operation, the patient had an uneventful recovery and was discharged home on POD 8. On the day after surgery, a contrast-enhanced CT scan was performed to assess the status of the hemangioma, and resolution of the lesion was confirmed (**Fig. 5**).

## DISCUSSION

In the setting of LDLT, hepatic hemangiomas have occasionally been identified in donor liver grafts.^5–7)^ In the reported cases, the hepatic hemangioma was located within the graft and excised either *in vivo* during donor surgery or *ex vivo* on the back table before graft implantation. These reports demonstrate that the presence of a hepatic hemangioma in a liver graft, when appropriately managed, does not preclude the safe use of the graft. However, the safety of performing a hepatectomy in a living liver donor with a hepatic hemangioma located in the remnant liver, such as in the present case, has not been discussed.

Rupture of a hepatic hemangioma is a rare but potentially life-threatening complication, with reported mortality rates of 60%–75% in the absence of prompt intervention.^10)^ Tumor size is considered as a key risk factor, with larger lesions carrying a higher likelihood of rupture.^11,12)^ Interestingly, the lesion in our patient was only slightly larger than 2 cm in diameter, well below the threshold typically associated with spontaneous rupture. Several mechanisms may have contributed to the rupture in this case. Possible precipitating factors include external stress from the surgical manipulation during hepatectomy and ischemia–reperfusion stress induced by the intermittent Pringle maneuver during the parenchymal transection. Although hand-assisted laparoscopic mobilization of the liver is generally considered a protective maneuver, external stress may have been applied to the hemangioma located relatively close to the liver surface in this case. Recently, Neto et al.^13)^ described the rupture of a relatively small hemangioma (2 × 3 cm) within a liver graft immediately after transplantation. In that case, similar potential mechanisms, including inherent coagulopathy in the early postoperative period after liver transplantation, ischemia–reperfusion injury, and surgical manipulation during either the donor or recipient procedure, were indicated. Therefore, while the presence of small asymptomatic hemangiomas is not generally considered a contraindication to donor hepatectomy, awareness of the potential but rare risk of rupture is warranted, particularly in the immediate postoperative period, when the remnant liver is subject to increased mechanical stress. Hepatic hemodynamic changes may be discussed as another possible mechanism for the rupture of a hepatic hemangioma in the remnant liver after hepatectomy. Nevertheless, given that hepatectomy usually results in increased portal venous inflow or portal venous pressure accompanied by decreased hepatic arterial flow,^14,15)^ it appears theoretically implausible that a hemangioma predominantly supplied by the hepatic artery would rupture due to such alterations.

Management options for a ruptured hepatic hemangioma include hemostasis via IVR, most commonly TAE, and surgical resection. TAE is widely regarded as a first-line treatment for hemangioma rupture, especially in hemodynamically stable patients.^16,17)^ In the present case, TAE was initially considered. However, surgical intervention was ultimately chosen for two main reasons. First, selective partial TAE was technically challenging, and non-selective embolization risked extensive hepatic infarction and postoperative liver failure. Second, the large intrahepatic hematoma was compressing the portal vein, raising concern for further compromise of the portal venous flow. In cases without prior hepatectomy, or in post-hepatectomy settings where the remnant liver volume is sufficient and the risk of liver failure is considered low, IVR can be considered a viable alternative. In the present case, intraoperative ultrasonography did not allow accurate identification of the hepatic hemangioma. Therefore, we judged that resection of the hemangioma would be difficult in this case and elected to proceed with evacuation of the hematoma, identification of the bleeding point, and extensive hemostatic suturing. If a residual hemangioma had been present, a staged resection would have been considered owing to the potential risk of rebleeding. However, the contrast-enhanced CT scan on the day after surgery showed that the hemangioma had become indistinct as a result of the extensive hemostatic suturing, and no additional surgical intervention was considered necessary.

## CONCLUSIONS

This case highlights a rare complication: rupture of a hepatic hemangioma in the remnant liver of a living liver donor following hepatectomy. Even small hepatic hemangiomas can rupture under specific postoperative physiological conditions. Surgeons should be vigilant for this rare but serious complication. Careful postoperative monitoring and a low threshold for imaging evaluation in the presence of anemia or abdominal discomfort may facilitate early detection and timely management of this rare complication in a living liver donor with a hepatic hemangioma in the remnant liver.

## DECLARATIONS

### Availability of data and materials

Data will be made available on reasonable request.

## References

[1] Mocchegiani F, Vincenzi P, Coletta M, et al. Prevalence and clinical outcome of hepatic haemangioma with specific reference to the risk of rupture: a large retrospective cross-sectional study. Dig Liver Dis 2016; 48: 309–14. 26514738 10.1016/j.dld.2015.09.016

[2] Sidler F, Turcan V, Storni F, et al. Spontaneous atraumatic rupture of a liver hemangioma as a rare cause of syncope. Case Reports Hepatol 2024; 2024: 7921410. 39104460 10.1155/2024/7921410 PMC11300087

[3] Nakabayashi R, Miyachi Y, Torai M, et al. Small hepatic hemangioma leading to life-threatening bleeding following blunt abdominal trauma: a case report. J Acute Care Surg 2023; 13: 134–7.

[4] Nguyen M, Lim AE, Jeyarajan E, et al. Giant hepatic haemangioma rupture in a patient on direct oral anticoagulant therapy. J Surg Case Rep 2021; 2021: rjaa523. 33542805 10.1093/jscr/rjaa523 PMC7850050

[5] Onishi Y, Kamei H, Imai H, et al. Successful adult-to-adult living donor liver transplantation using liver allograft after the resection of hemangioma: a suggestive case for a further expansion of living donor pool. Int J Surg Case Rep 2015; 16: 166–70. 26476494 10.1016/j.ijscr.2015.09.043 PMC4643476

[6] Li SX, Tang HN, Lv GY, et al. Pediatric living donor liver transplantation using liver allograft after ex vivo backtable resection of hemangioma: a case report. World J Clin Cases 2022; 10: 3834–41. 35647153 10.12998/wjcc.v10.i12.3834 PMC9100736

[7] Pacheco-Moreira LF, Enne M, Balbi E, et al. Hemangioma at the liver section plane. Is it a cintraindication for living donor liver transplantation? Surgery 2005;138:113. 16003328 10.1016/j.surg.2005.03.027

[8] Eguchi S, Soyama A, Hara T, et al. Standardized hybrid living donor hemihepatectomy in adult-to-adult living donor liver transplantation. Liver Transpl 2018; 24: 363–8. 29194959 10.1002/lt.24990

[9] Soyama A, Takatsuki M, Adachi T, et al. A hybrid method of laparoscopic-assisted open liver resection through a short upper midline laparotomy can be applied for all types of hepatectomies. Surg Endosc 2014; 28: 203–11. 23982655 10.1007/s00464-013-3159-1

[10] Ribeiro MA Jr, Papaiordanou F, Gonçalves JM, et al. Spontaneous rupture of hepatic hemangiomas: a review of the literature. World J Hepatol 2010; 2: 428–33. 21191518 10.4254/wjh.v2.i12.428 PMC3010512

[11] Donati M, Stavrou GA, Donati A, et al. The risk of spontaneous rupture of liver hemangiomas: a critical review of the literature. J Hepatobiliary Pancreat Sci 2011; 18: 797–805. 21796406 10.1007/s00534-011-0420-7

[12] Brouwers MA, Peeters PM, de Jong KP, et al. Surgical treatment of giant haemangioma of the liver. Br J Surg 1997; 84: 314–6. 9117293

[13] Neto JS, Travassos NPR, Costa CM, et al. Rupture of hepatic hemangioma following living donor liver transplantation: a case report. Pediatr Transplant 2025; 29: e70122. 40589300 10.1111/petr.70122

[14] Golriz M, El Sakka S, Majlesara A, et al. Hepatic hemodynamic changes following stepwise liver resection. J Gastrointest Surg 2016; 20: 587–94. 26573852 10.1007/s11605-015-3021-y

[15] Bogner A, Reissfelder C, Striebel F, et al. Intraoperative increase of portal venous pressure is an immediate predictor of posthepatectomy liver failure after major hepatectomy: a prospective study. Ann Surg. Ann Surg 2021; 274: e10–7. 31356261 10.1097/SLA.0000000000003496

[16] Torkian P, Li J, Kaufman JA, et al. Effectiveness of transarterial embolization in treatment of symptomatic hepatic hemangiomas: systematic review and meta-analysis. Cardiovasc Intervent Radiol 2021; 44: 80–91. 32808203 10.1007/s00270-020-02611-5

[17] Cao Y, Xiong F, Xiong B, et al. A case of spontaneous hepatic hemangioma rupture: successful management with transarterial chemoembolization alone. J Interv Med 2019; 2: 131–3. 34805887 10.1016/j.jimed.2019.09.014 PMC8562225

